# What Is Safe Limit of the Perforator Flap in Lower Extremity Reconstruction? Do We Have Answers Yet?

**DOI:** 10.1155/2011/349357

**Published:** 2011-10-11

**Authors:** Nikhil S. Panse, Yogesh C. Bhatt, Mangesh S. Tandale

**Affiliations:** ^1^Department of Plastic Surgery, B.J. Medical College, Pune 411004, India; ^2^Vimal Niwas, Sudarshan Society, Near Model Colony Post Office, Shivajinagar, Pune 411016, India; ^3^Department of Plastic Surgery, SSG Hospital & Medical College, Baroda 390001, India; ^4^Department of Plastic Surgery, Citicare Hospital, Aurangabad 431001, India

## Abstract

We make an attempt to define the safe extent of local perforator flap for lower limb reconstruction by comparing it with the limb length of the patient. The maximum flap length from the perforator was compared to the limb length in 35 patients using EPI info 6.04 D software. On comparison of flaps that were less than one-third of limb length to those which were more than one-third of limb length, the statistical values were significant. The odds ratio calculated was 6, which means that there is a six times more chance that a local perforator flap will necrose if it is more than one-third of the limb length as compared to a flap which is less than one-third of the limb length.

## 1. Introduction

Lower limb trauma is one of the most commonly encountered problems by the plastic surgeon. Various modalities of options from locoregional to free flaps have been described for lower extremity defect reconstruction [1].

 French and Tornetta [1] review the recent literature of lower extremity trauma and options for bone fixation and soft tissue coverage.

 Fasciocutaneous flaps are difficult to reach in the distal part of the leg. Musculocutaneous flaps lead to loss of function of the particular muscle. Free flaps are considered to be very good options, but the obvious drawbacks are that it needs considerable expertise and generally one of the two major vessels is used for anastomosis. 

So, there has been considerable stress to find out local flap coverage for these defects, a flap which would be technically easier than the free flaps, which would not lead to loss of function of any muscle or muscle groups, can be used in all parts of the leg, have minimum donor site morbidity, and is reliable. Perforator flaps are the nearest to the above-mentioned criteria. However, there are no studies to define the safe limit of the perforator flap in lower extremity reconstruction. An attempt is made to refine the technical operative details of the perforator flap and to define the safe limit of the perforator flap in lower extremity reconstruction. 

## 2. Material and Methods

Thirty-five patients of lower limb defects below the knee were considered in the study. Only those patients where the perforator flap was islanded were included. Perforator plus flaps where the base of the flap was kept intact were not included in the study. The details of the operative procedure are as follows.

We did preoperative perforator identification by hand-held Doppler with an 8 Hz probe in all the patients. All the perforators along the surface markings are marked, and the axis of the flap is marked in between the perforators. The surface marking of vascular axis of all the three main vessels of the leg is as follows.

**For Posterior Tibial Artery**

A reference line is drawn by joining the tibial tuberosity and the midmalleolar point. The vascular axis lies approximately 4.5 cm medial and parallel to this line or 1.5 cm from medial border of tibia [2].

**For Anterior Tibial and Peroneal Arteries**

The reference line is drawn by joining the head of fibula and tip of lateral malleolus. The anterior tibial artery axis lies 2.5 cm anterior and parallel to this reference line, and peroneal artery axis lies 2.5 cm posterior and parallel to this reference line [2].

Debridement and flap elevation is done under tourniquet control. While inflating the tourniquet, the popliteal veins are blocked so as to prevent complete venous exsanguination of the limb. Some amount of venous stasis helps in perforator identification and dissection. 

 The width of the flap depends on the width of the defect and the angle of rotation of the flap. The width of the flap equals the vertical height of the defect if the flap is rotated 90 degrees, while it equals the anteroposterior diameter of the defect in case of 180-degree rotation. The flap can be extended posteriorly up to the midline of the calf.

**Figure 1:**
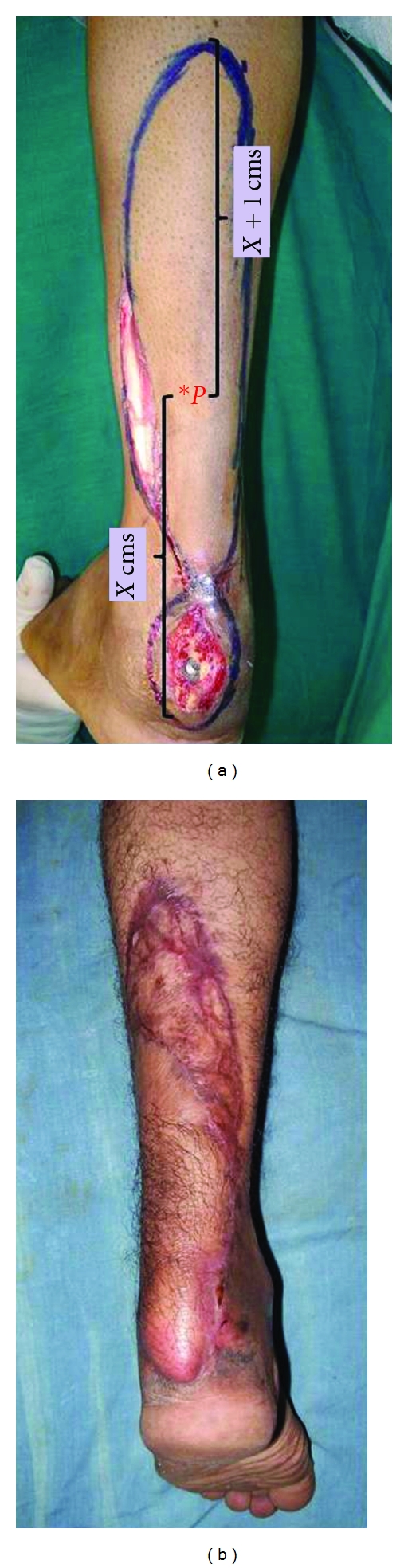


 The superior margin of the flap is determined intraoperatively, after mobilization of the pivot perforator (*P*). The length of the flap proximal to the pivot perforator (*P*) equals the length from (*P*) to the edge of the defect to be closed plus 1 cm. This 1 cm is added to allow tension-free closure (Figure 1).

After excision of the ulcer, the posterior incision is made through skin and deep fascia. Because dominant or primary cutaneous arteries emerge from the deep fascia near where the fascia is fixed to bone or anchored by intermuscular septa, these sites, as revealed by natural skin crease lines, are explored first. Subfascial sharp dissection proceeds anteriorly until the intermuscular septum is reached. Sharp dissection preserves the subfascial plexus. This plexus is less important than the suprafascial one, but we feel that it has a role in the blood supply of the deep fascia. On reaching the intermuscular septum, the perforators are exposed and mobilized by dissecting the septum. 

The pivot perforator is completely mobilized, and its integrity is determined. The anterior incision is opened and the proximal end of the flap is measured and cut. At this stage, the flap is attached to the leg by the perforators only, 3–5 in number. All the extra perforators are clamped with microclamps. The viability of the flap, shown by refilling of vessels and bleeding from the flap, is observed. If viability is judged good, the clamped perforators are ligated and cut.

 A reliable perforator is believed to be having the ability to expand its perfusion over its territory after perforator flap elevation. Based on the clinical experience, the reliable is a perforator that sprouts from carrier muscles or septum with a visible pulsation. It has the peculiar ability to overcome the angiosome barrier through subdermal network. The perforators that are not to be used are cut after dissecting for an adequate length, so that they can be used for supercharging the flap in case of vascular compromise.

Now, the flap is an island attached only by its pivot perforator artery and its venae comitantes. The flap is rotated on the axis of this perforator up to 180 degrees to cover the defect. After rotation, the viability of the flap is reassessed. If there is any compromise, there may be some fibers of the septum remaining around the pedicle which are compressing the perforator or the pedicle may not be completely mobilized to the donor vessel. In such cases, further dissection is done to ensure that the pedicle vessels are completely mobilized. The perforator dissection to their origin from the donor vessel is not done in all cases. It is only done in cases where there is inadequate mobility and torsion on the perforator due to its short length.

**Figure 2:**
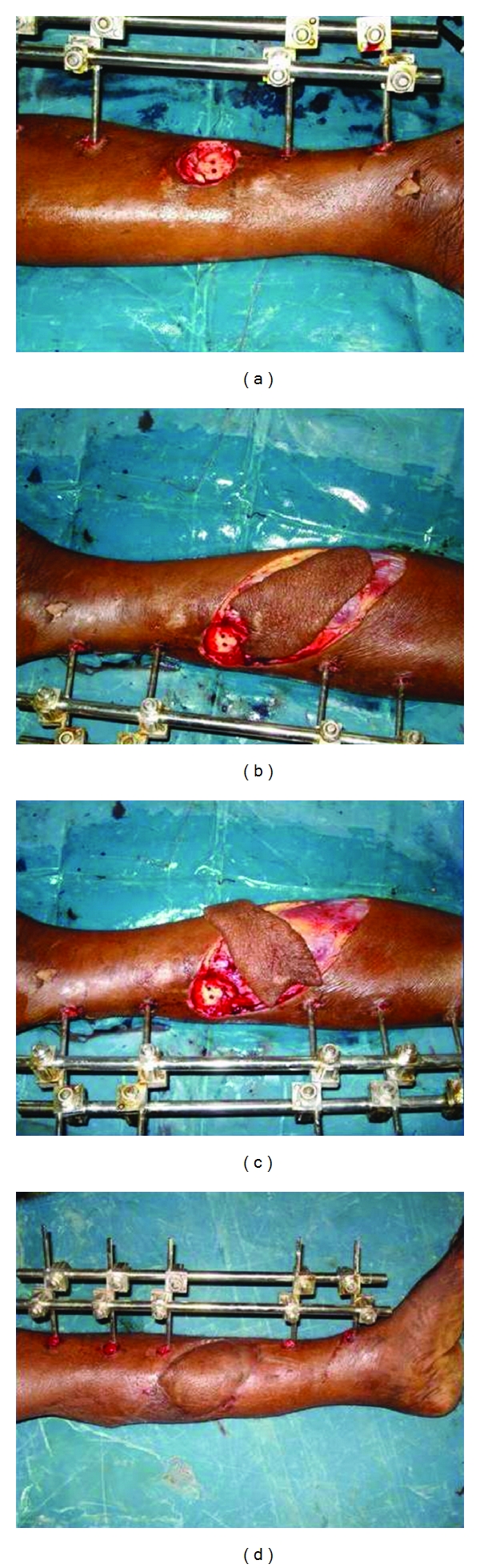


**Figure 3:**
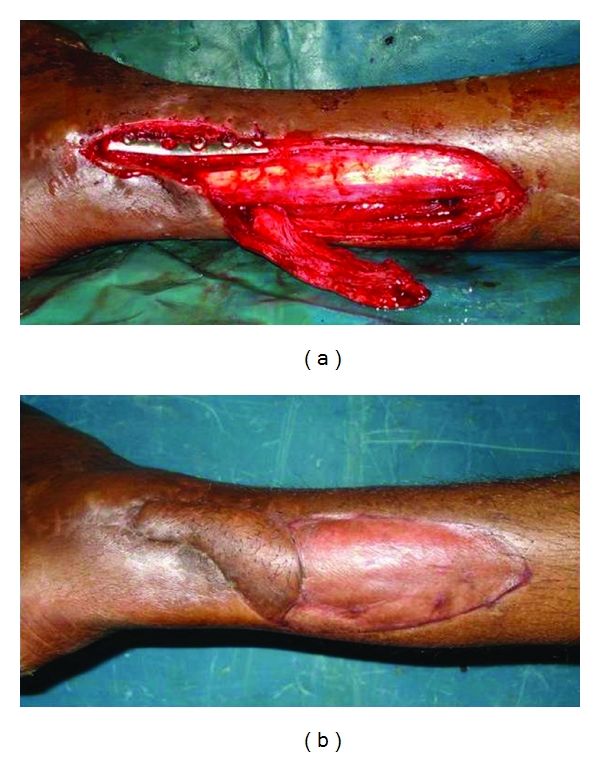


**Figure 4:**
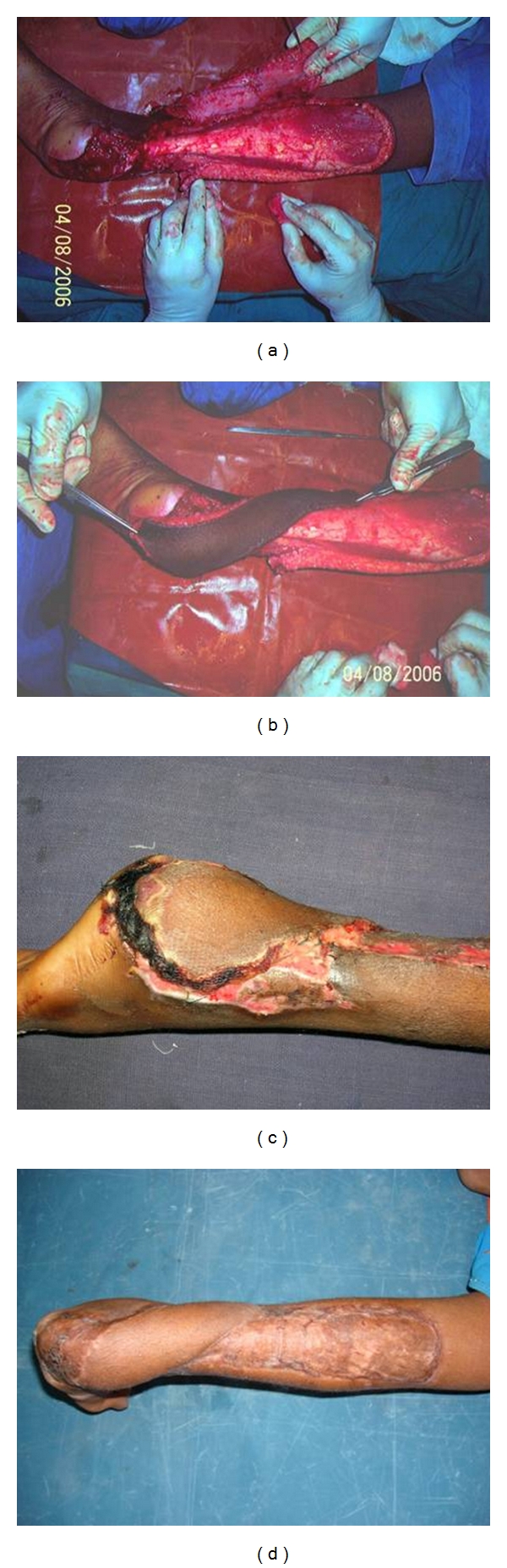


**Figure 5:**
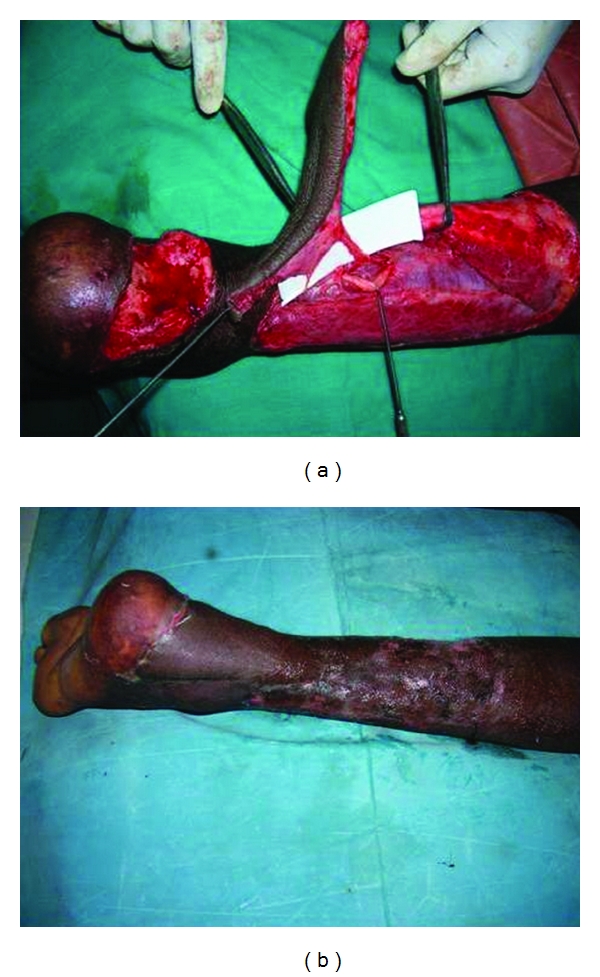


 The flap is sutured in its new position, and the donor area is covered by split skin graft. The initial two sutures are placed on the side of the perforators so as to prevent torsion and stretch on the perforator. Stretching and drying are the most common causes of perforator thrombosis during the operation and must be prevented. Twisting of the pedicle occurs more commonly in perforator flaps than in conventional flaps. Because there is no muscle accompanying the pedicle as it enters the skin, it can twist around. Inclusion of a small muscle cuff in case of musculocutaneous perforators at the entrance of the perforator to the flap to prevent twisting and paying special attention to the course of the vessels during the inset are vital maneuvers. Light dressings cover the flap, which can be monitored easily for any change in color (Figures 2, 3, 4, and 5).

A posterior splint keeps the leg extended and immobilized for ten to twelve days postoperatively. The foot is kept elevated on one or two pillows during this period. Pressure dressings in the form of crepe bandage are started from postoperative day seven.

**Important Technical Considerations in our Study**

Perforator flap dissection is more often all about patience than about skills.

- Preoperative marking of the dominant perforator.
- Wide exposure of the surgical field and bloodless dissection are the keys.
- Skin island must be centered on the top of the perforator or following the direction of the main branch of the perforators within the flap.
- Dissect the perforators under loupe or microscopic magnification.
- Avoid drying and spasm by constant irrigation with lignocaine and saline solution.
- Thin rim of fat can be left around the perforator for additional support and to prevent kinking.
- However, all fibrous strands have to be dissected and the perforator denuded to prevent kinking due to any strand.
- The initial sutures that are to be taken when suturing the perforator flap in place are the two sutures on both sides of the perforator so that there is no kink and torsion on the pedicle.
- Before transection of the extra perforators, the most dominant perforator is to be identified by clamping the perforators.
- Flap is inset without tension to avoid circulatory disturbances at the distal part.

Using EPI info 6.04 D software analysis of the results was done, and various tests were performed as needed.

## 3. Observation and Results

Total of thirty-five consecutive patients operated from August 2005 till January 2008 were included in the study. Followup of patients ranged from 30 days to one year. All the defects included in the study were below knee and of traumatic origin. All the flaps included in the study were based only on a single perforator and islanded.

The minimum age at which flap was done was 6 years, and the maximum age of the patient was 60 years. The average age of the patients was 32 years. There were 28 male patients and 7 female patients. The average time since trauma when the patient was operated upon was 18 days. The average maximum distance of the flap from the perforator was 12 cms. The average limb length measured was 38 cms. The average time taken for surgery was two hours and fifteen minutes. The average ward stay in the postoperative period was 14 days. Of the defects, two defects were in the upper third, ten in the middle third, and twenty-three in the lower third. Of the 35 perforators, 28 were septocutaneous and 7 were musculocutaneous. Four were perforators from the anterior tibial, 18 from the posterior tibial, and 13 from the peroneal vessels (Table 1).

**Figure 6:**
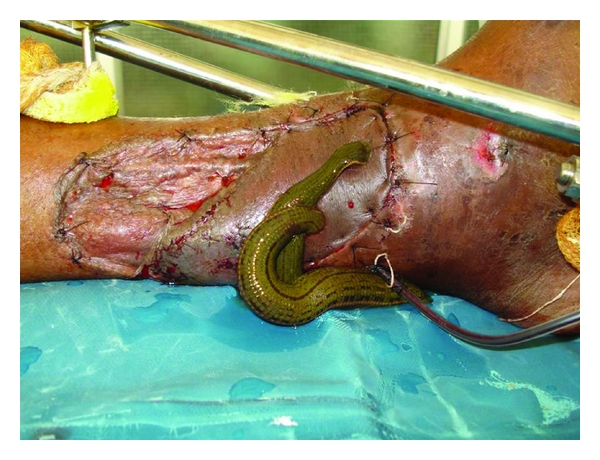


There was complete flap loss in 3 patients and partial flap loss in 7 patients. One patient had an osteomyelitic sinus, and one patient had graft loss, which required grafting. All the flaps were lost due to congestion. Salvage of one congested flap was attempted by leech application; however, it could not be salvaged (Figure 6). Two patients with complete flap loss were managed by cross-leg flap and grafting, respectively. The third patient of complete flap loss absconded from the ward and was lost to followup. Inferolaterally based fasciocutaneous flaps were done for two patients with marginal flap necrosis. One patient with marginal flap necrosis was grafted. The remaining four patients with marginal flap necrosis healed secondarily. One patient developed osteomyelitic sinus and is in followup with our orthopedic colleagues and is currently being managed conservatively.

The main complication that is flap necrosis, complete or partial, was studied in relation to the age, time since trauma, type of the flap that is perforator or propeller, the Gustillo Anderson fracture classification, and the limb length. On statistical analysis, only the limb length was found to be statistically significant.

**Flap Necrosis in Relation to Limb Length**

The maximum distance of the flap from the perforator was measured and compared to the limb length. The limb length was measured from the superolateral aspect of the lateral malleolus to the fibular head in all the patients. Analysis was made by dividing the flaps into two groups, first group where the maximum flap length was less than one-third of the limb length and second group where maximum flap length was more than one-third of the limb length.

Fisher exact test was used for the analysis. The 1-tailed *P* value was 0.0291485 and 2-tailed *P* value was 0.0351426. Both of the values were less than 0.05 and were statistically significant. 

The odds ratio calculated was 6, which means that there is a six times more chance that a local perforator flap will necrose if it is more than one-third of the limb length as compared to a flap which is less than one-third of the limb length (Table 2).

## 4. Discussion

A variety of perforator flaps have been described based on the perforator of anterior tibial, posterior tibial, and the peroneal vessels. It was proposed that the safe extent of the perforator flap was the distance between the two perforators [3–5]. However, the distance between two perforators is not constant and it has to be determined intraoperatively as to how much flap is to be harvested. As of now, there are no studies to define the safe extent of the perforator flap.

 Whenever the defect size and the vascular condition of the neighboring tissues allow a reconstruction with local perforator flaps, the surgical intervention and the morbidity ought to be limited to a single body region. The early designs of the local flaps along the vertical axis of the leg or the thigh with a proximal pedicle have been modified by the use of perforator flaps.

We know that in normal systemic circulation vessel diameter decreases towards periphery, but, because vessels branch widely like a tree, the area of the section of the aorta is smaller than the whole area of the section of a more distal segment [2, 6].

 From a haemodynamic point of view, this means that blood velocity decreases from aorta to peripheral blood vessels and that flow is divided in each of the branching vessels, as in a hydraulic in parallel system [2, 6]. 

 In a perforator flap, all branching vessels are closed, apart from a skin perforator, and, therefore, from the origin of the pedicle to the skin, there is a single conduit with decreasing diameter. In this condition, we know from physics, assuming a non-Newtonian liquid with rigid walls, that all the liquid that enters the conduit should get out of it and velocity is higher in sections with smaller diameter [6, 7]. 

Studies have shown that, in normal anatomic condition, blood velocity in the perforator is lower than that in the corresponding pedicle, whereas after surgery, that is, in perforator flap architecture, blood velocity in the perforator is higher than that in the corresponding pedicle. Therefore, there is an inversion of the gradient of blood velocity between the pedicle and the perforator compared to normal circulation, a phenomenon that is called the “inversion of velocity gradient” in perforator flaps.

As to flow rate, in normal circulation flow through the perforator is much smaller than that at the pedicle, while after surgery, in the flap, flow through the perforator is still smaller but is a much greater proportion of the flow through the pedicle [6]. 

The blood supply to skin in perforator flaps is increased, being a big proportion of the blood flow through the pedicle artery [6]. In the clinical setting, this means that perforator flaps can replace muscle flaps where the use of muscle flaps is indicated merely because of their abundant blood supply and not for restoring function [6, 7].

In a clinical setting, there are multiple other factors which can influence the outcome of the perforator flap like size of the particular perforator and its ability to overcome the particular angiosome, distance between two perforators, associated vascular disease, trauma zone, posttraumatic vascular disease of the vessel and the perforator, spasm of the perforator at that particular time because of systemic factors or surrounding temperature or handling of tissues. It is because of multiple factors that it is difficult to predict with certainty the safe limit of a perforator propeller flap in lower extremity reconstruction. It is our observation that perforator propeller flaps with maximum flap length less than or equal to one-third of limb length are safe flaps. However, we do not claim it to be a gold standard formula for decision making in lower extremity reconstruction using perforator flaps. It is a rough guideline to be considered along with various other factors mentioned above for optimal outcomes.

In conventional local fasciocutaneous flaps, the act of rotating the tissue results in a twist at the base of the flap and a twisting of the pedicle in an islanded flap. This in turn results in an increase in external pressure on the perforating vessels as they twist with the tissue, which is more pronounced in islanded flaps for equivalent arcs of rotation, as the torsional force is centered on the vascular pedicle [8]. The length of vascular pedicle is also significant, as a greater torsional force will be transmitted to shorter pedicles, for a given degree of rotation [8]. It is therefore necessary for complete dissection and mobilization of the perforator to decrease the torsional forces compromising the vascularity of the perforator flap [8]. A further consideration is that thinner-walled veins with lower vessel wall elasticity, and lower intraluminal pressure, are more sensitive to torsional forces compared to the arteries [8, 9].

**The main advantages of these flaps are as follows.**

- They are easy to learn.
- No special instruments are necessary—the preoperative Doppler may be helpful but is not essential.
- They do not involve sacrifice of one of the main leg arteries.
- They can cover very distal defects of the leg.
- The donor site has muscle in its base and is reliably resurfaced with a skin graft.
- Donor site morbidity is limited to a single body area, and use of propeller flaps has concretely widened the reconstructive options for lower extremity reconstruction.
- There is an important decrease in the donor site morbidity by preserving the muscle and nerve function.
- There is a specific like to like soft tissue replacement leading to a better cosmetic and reconstructive outcome.
- The operative time taken for perforator propeller flaps is not significantly higher than that for fasciocutaneous flaps.
- The perforator flap has nothing to do with the notorious anatomical variations. One has only to identify a reliable perforator and isolate it irrespective of the anatomic variations of the donor vessel.
- Exclusion of the muscle from the perforator flap makes it a more pliable flap because of the absence of fibrosis of muscle in long-term followups.
- Perforator propeller flaps form an important substitute for muscle flaps for providing excellent blood supply to the recipient area along with preservation of the muscle.

## 5. Conclusion

In our study of thirty-five perforator propeller flaps, an attempt was made to define the safe extent of the perforator propeller flap. We analyzed our complications with regard to the age group of the patient, the postinjury days, the area of the defect, the type of the perforator, the donor vessel, the associated fracture type, and the type of flap that is perforator or propeller. We have measured the maximum flap length from the perforator and have compared it to the limb length of the patient. 

On analysis of the data, we have concluded that perforator propeller flaps with maximum flap length equal to or less than one-third of the limb length are safe flaps. However, it is not gold standard formula for decision making in lower extremity reconstruction using perforator flaps. We suggest that it should be considered as a rough guideline along with various other factors for optimal outcomes.

 Whenever the expected maximum flap length, limb length ratio exceeds more than one-third, and it is better to look for other reconstructive options. This flap is a simple, safe, and versatile procedure to cover moderate-sized traumatic lower extremity wounds.

## Figures and Tables

**Table 1:** 

Sr. no.	Site of defect	Gustillo classification/soft tissue defect	H/o tobacco	Time since trauma (days)	Distal flap extent from perforator—flap length (cm)	Leg length (cm)	Ratio of flap to leg length (%)	Distance from bony landmark (cms)
1	L3rd	III A	Yes	29	9	38	23.68	13
2	L3rd	II	Yes	21	14	39	35.89	11
3	L3rd	II	Yes	16	11	39	28.2	13
4	L3rd	III A	Yes	13	11	38	28.94	13
5	M3rd	II	Yes	33	13	36	36.11	13
6	L3rd	II	Yes	45	9	39	23.07	11
7	L3rd	III A	Yes	16	8	38	21.05	5.5
8	L3rd	III A	Yes	7	13	39	33.33	13
9	L3rd	III A	No	15	14	36	38.88	12
10	L3rd	I	Yes	21	9	36	25	8
11	M3rd	III A	No	30	13	38	34.21	20
12	L3rd	Heel pad defect	No	3	17	40	42.5	5
13	M3rd	II	Yes	14	13	39	33.33	22
14	U3rd	III A	Yes	9	9	38	23.68	24
15	L3rd	III A	Yes	17	14	39	35.89	13
16	M3rd	II	Yes	7	8	38	21.05	20
17	L3rd	Tendoachillis defect	Yes	4	13	39	33.33	5
18	L3rd	Tendoachillis defect	No	15	12	39	30.76	5
19	M3rd	III A	Yes	8	11	39	28.2	18
20	L3rd	II	Yes	12	9	40	22.5	5
21	L3rd	II	No	30	11	38	28.94	8
22	L3rd	II	No	14	7	22	31.81	12
23	M3rd	II	Yes	8	8	39	20.51	7
24	M3rd	II	Yes	10	8	38	21.05	15
25	L3rd	III A	Yes	24	13	38	34.21	11
26	L3rd	II	No	15	10	26	38.46	8
27	L3rd	II	Yes	18	13	40	32.5	13
28	L3rd	III A	Yes	8	6	38	15.78	15
29	L3rd	III A	No	30	17	36	47.22	5
30	M3rd	II	Yes	15	7	38	18.42	24
31	L3rd	II	Yes	27	10	38	26.31	12
32	U3rd	II	Yes	21	8	38	21.05	28
33	M3rd	II	Yes	13	6	38	15.78	13
34	L3rd	II	No	18	10	37	27.02	8
35	M3rd	II	Yes	14	8	38	21.05	11

**Table 2:** 

Percentage of flap compared to limb length	Total flaps	Necrosis	Percentage
<33.33% (1/3rd of limb length)	21	2	9.52
>33.33% (1/3rd of limb length)	14	8	57.14

Total	35	10	28.57
